# Concurrent Perioperative Diagnosis of HIV in a Patient With Plunging Ranula: A Case Report

**DOI:** 10.7759/cureus.44832

**Published:** 2023-09-07

**Authors:** Mohd Faizal Abdullah, Shaifulizan Abdul Rahman, Fattirah Auni Fauzi

**Affiliations:** 1 Oral and Maxillofacial Surgery, Hospital Universiti Sains Malaysia, Kota Bharu, MYS; 2 Oral and Maxillofacial Surgery, School of Dental Sciences, Universiti Sains Malaysia, Health Campus, Kota Bharu, MYS

**Keywords:** salivary gland, surgical excision, hiv-related oral lesion, plunging ranula, hiv

## Abstract

Oral manifestations may be the earliest indicators of HIV infection as it has strong association with oral candidiasis, hairy leukoplakia, linear gingival erythema, necrotizing ulcerative gingivitis, necrotizing ulcerative periodontitis, Kaposi sarcoma, and lymphoma. Other conditions such as diffuse infiltrative lymphocytosis syndrome, benign lymphoepithelial cyst, and salivary gland neoplasm have also been reported in HIV patients. Ranulas are caused by salivary leakage from the sublingual gland as a result of ductal obstruction or trauma. At the present time, there is no clear evidence of a link between plunging ranula and HIV. The authors described a case of plunging ranula of the right floor of the mouth with a concurrent perioperative diagnosis of HIV. Surgical excision of ranula and associated salivary glands via submandibular and intraoral approach was successfully done with no recurrence over a period of one year. This case also highlights the importance of taking a thorough clinical history from patients and always practicing universal precautions, especially during surgical interventions.

## Introduction

Oral cavity affliction is a common early sign of HIV infection [1]. Salivary gland infiltrations are one of the most frequent oral presentations in HIV-positive individuals, and the parotid gland is the most commonly affected salivary gland [1,2]. Salivary gland infiltrations can manifest as both neoplastic and non-neoplastic diseases. Kaposi sarcoma and salivary gland lymphomas are examples of neoplastic diseases. Benign lymphoepithelial lesions, diffuse infiltrative lymphocytosis syndrome, and parotid lymphadenopathy are examples of non-neoplastic diseases [2].

Ranulas or retention cysts have also been described in association with HIV infection, but their association is poorly understood, especially when these lesions present as initial symptoms of HIV infection in the young age group. The HIV-related salivary gland disease includes various salivary gland lesions that frequently occur associated with HIV infection. The link between oral ranula and HIV has recently become more widely recognized [3]. According to some authors, the occurrence of ranula is one of the signs of HIV infection [4], and the study by Syebele et al. reports the observation of HIV1 virus in the fluid content of ranula in HIV-infected patients [5].

The majority of reported cases of ranula in HIV-positive patients came from Africa's Sub-Saharan region [4,6]. However, the reason for this specific geographical region remains unknown, and limited epidemiologic data from other continents revealed a poor associative linkage of the HIV-related salivary gland disease.

## Case presentation

**Figure 1:**
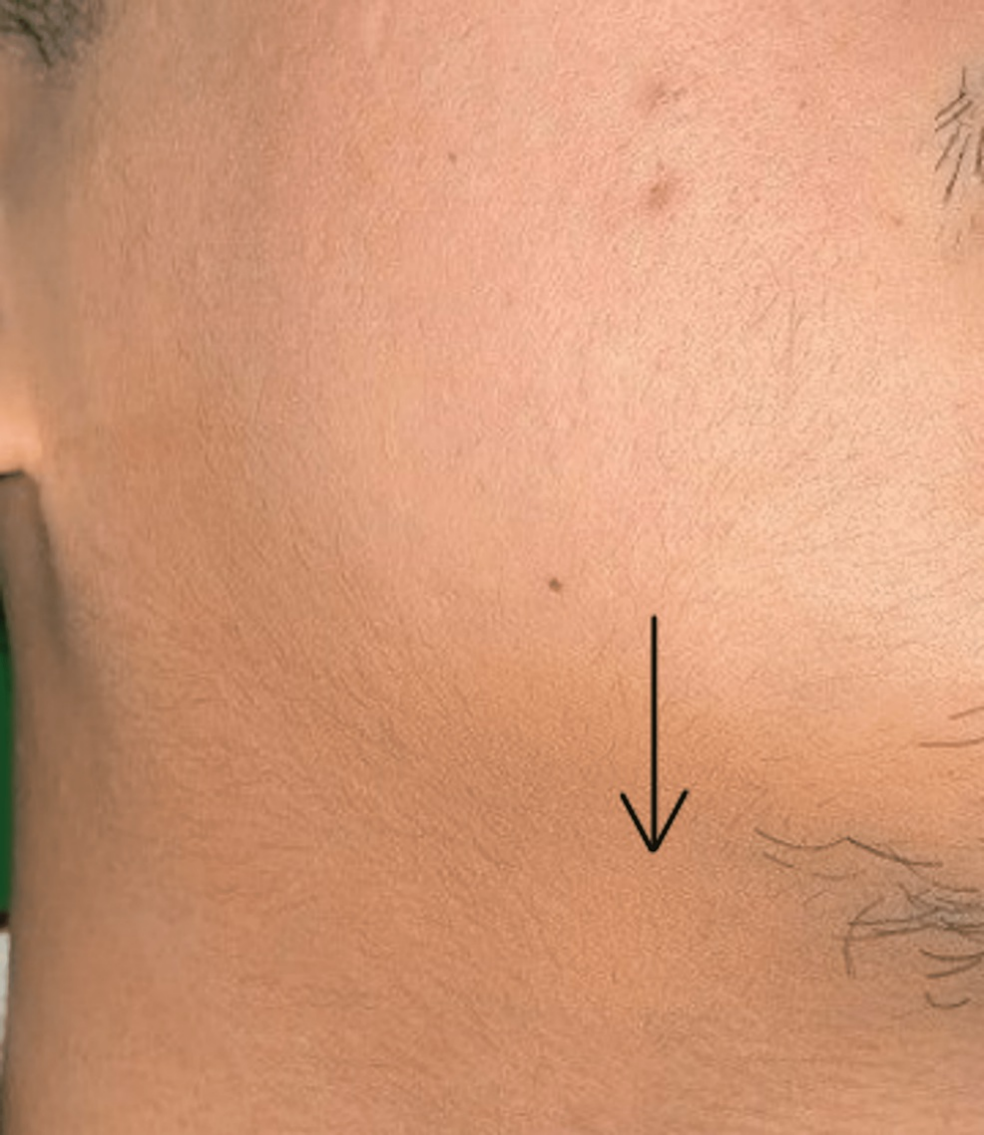
Extra oral photograph revealing minimal swelling over right neck region.

A 23-year-old patient presented with on and off swelling at the right submandibular region for a year, with pea-sized swelling at the right submandibular region and no intraoral swelling (Figure 1).

**Figure 2:**
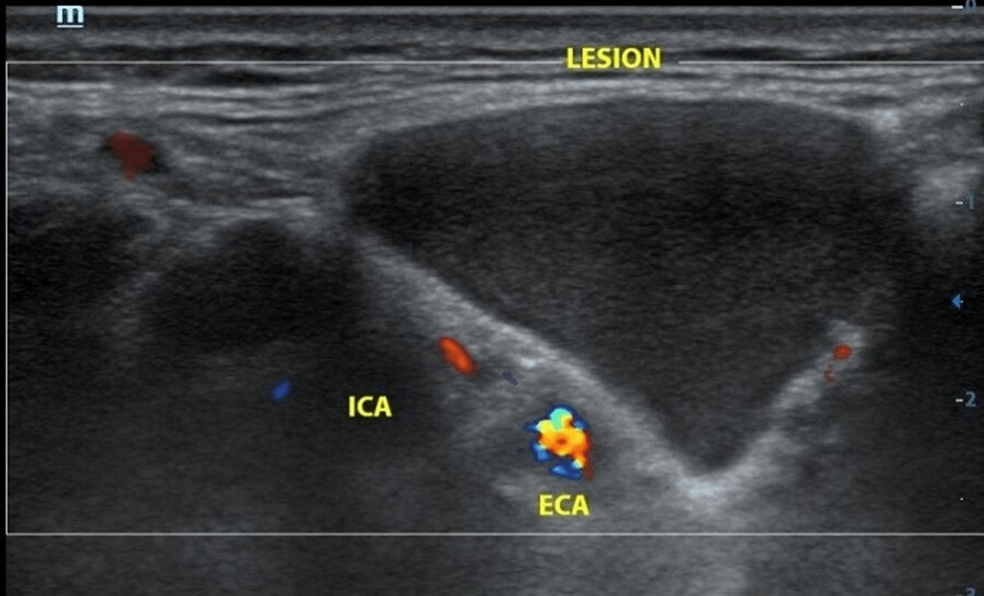
Anechoic lesion at the right submandibular region adjacent to right submandibular gland and medial to the carotid arteries. ECA: external carotid artery; ICA: internal carotid artery

Antibiotics were used to successfully treat one episode of infective swelling in the submandibular region. An ultrasound of the neck revealed a 4.3 cm x 2.0 cm x 5.1 cm anechoic lesion. Within it, there was debris and no septation, calcification, or vascularity; the differential diagnosis was a branchial cleft cyst or epidermal cyst (Figure 2).

**Figure 3:**
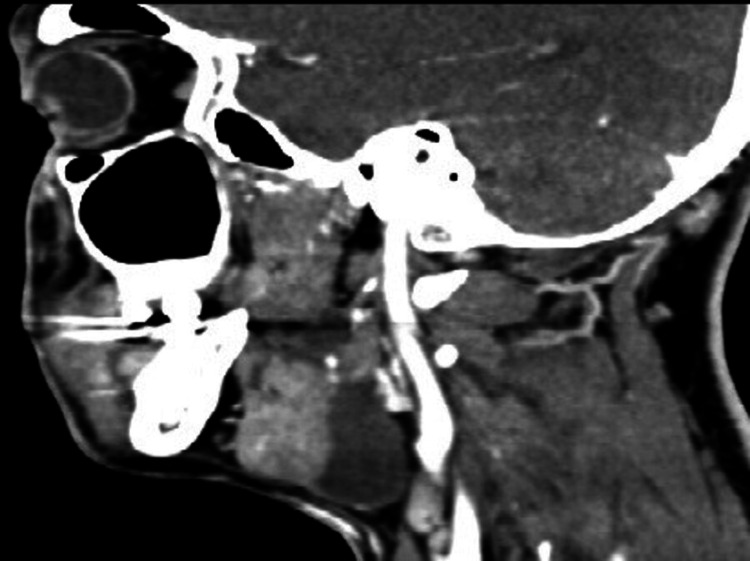
CT sagittal view of lesion showing posterior inferior extension abutting the right carotid artery and close relation to right submandibular gland.

A contrast-enhanced computed tomography (CECT) scan revealed a well-defined, non-enhancing, thin-walled cystic lesion at the sublingual space measuring 1.6 cm x 3.0 cm x 4.2 cm, insinuating between the mylohyoid muscle laterally, right medial pterygoid muscle and genioglossus muscle (medially), and extending into the right submandibular space (posteroinferiorly) confirming the clinical lack of intraoral swelling at this stage (Figure 3).

**Figure 4:**
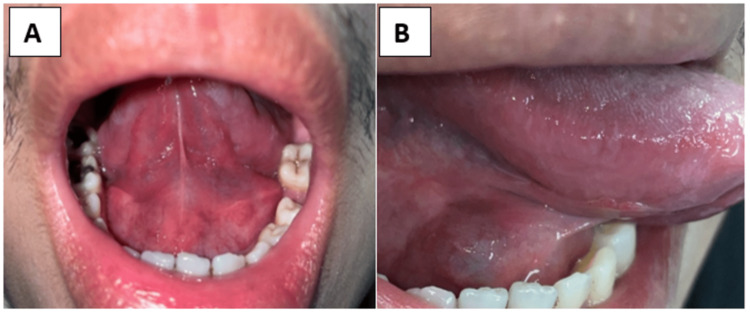
White lesion at the bilateral lateral border of tongue. Cystic lesion containing saliva-like fluid at both sides of floor of the mouth.

**Figure 5:**
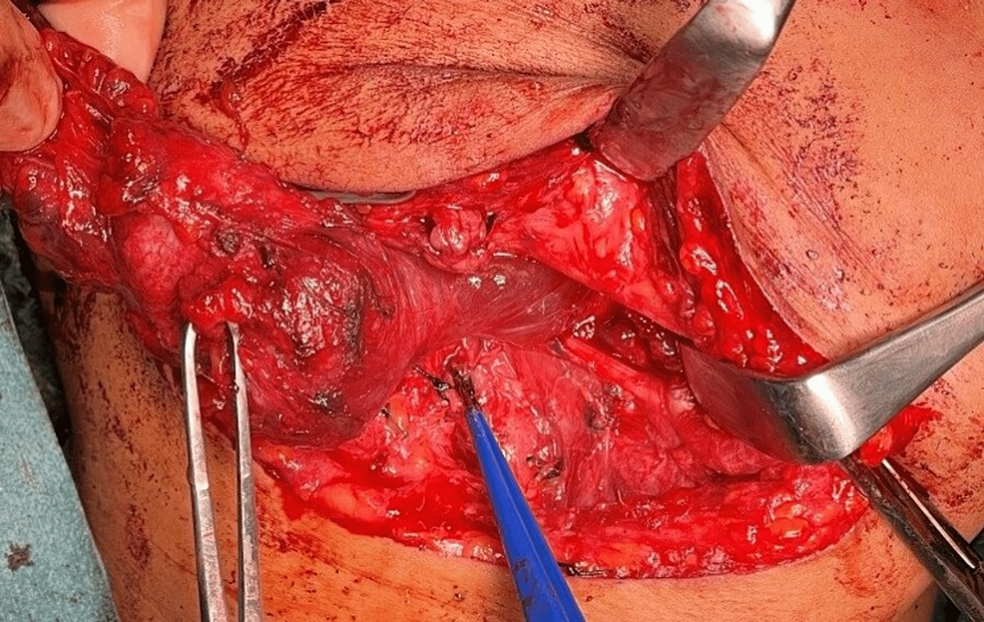
Removal of lesion via right submandibular and intraoral approaches.

**Figure 6:**
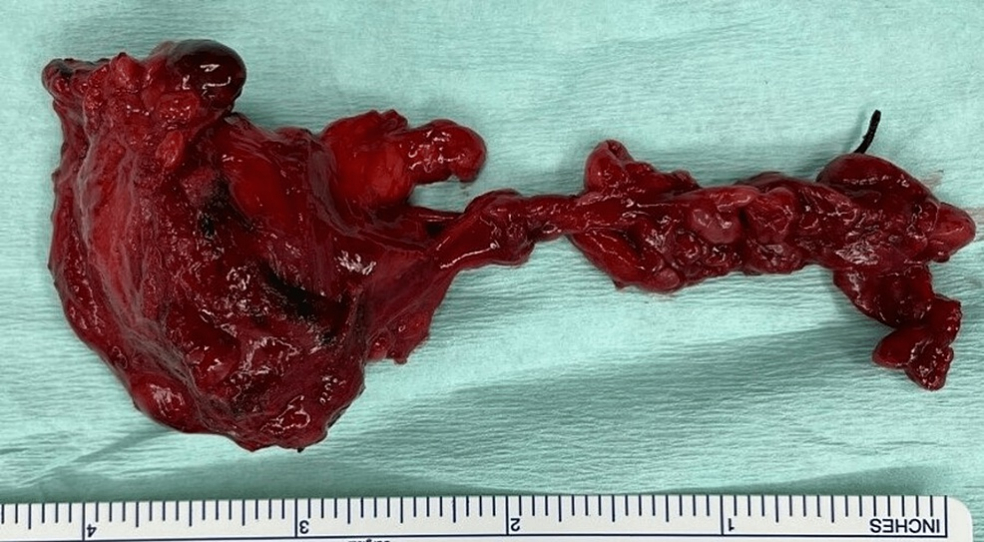
En bloc removal of lesion with right submandibular and sublingual gland.

Two weeks later, the patient complained of swelling on the right and left floor of the mouth, as well as a scrapable white lesion on the bilateral lateral border of the tongue (Figure 4). Oral swab specimen for culture analysis confirmed the presence of *Candida* infection. Intra-operatively, cystic lesion containing saliva-like fluid was noted (Figure 5) and the right submandibular gland, ranula, and right sublingual gland were removed (Figure 6). Histopathological examination (HPE) result revealed that the lesion was consistent with ranula.

The most difficult aspect of this case was obtaining an adequate history from the patient regarding his social life because the patient initially denied any high-risk behavior, but the finding of bilateral white lesions at the lateral border of the tongue was suspicious. However, after consultation and explanations to the patient regarding the possible association of ranula and white lesion with infective diseases, the patient agreed for infective screening test. On the third postoperative day, the infective screening test result confirmed that the patient was HIV-positive (Table 1).

**Table 1:** Infective screening test result. HBsAg: hepatitis B surface antigen; ECLIA: electrochemiluminescence immunoassay; HCV: hepatitis C virus; RPR: rapid plasma reagin; TPPA: Treponema pallidum particle agglutination *INNO-LIA™ HIV I/II Score assay: Fujirebio, Tokyo, Japan

Test	Result
HBsAg by ECLIA	Non-reactive
Anti-HCV by ECLIA	Non-reactive
Syphilis by ECLIA	Reactive
Titer syphilis ECLIA	294.1
Syphilis by RPR	Weakly reactive
Syphilis by TPPA	Positive
Comment on syphilis	Past or present syphilis history
HIV Ag/Ab by ECLIA	Repeatedly reactive
*INNO-LIA™ HIV	Positive

**Figure 7:**
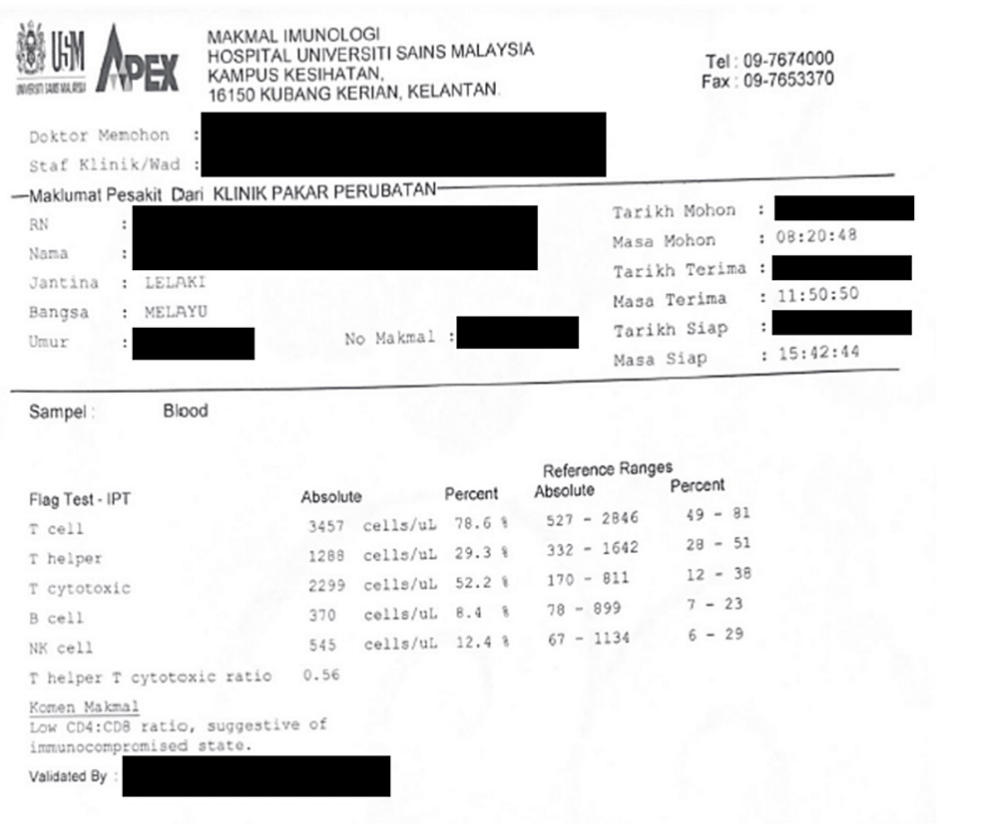
Report from immunology laboratory before HAART HAART: highly active antiretroviral therapy

**Figure 8:**
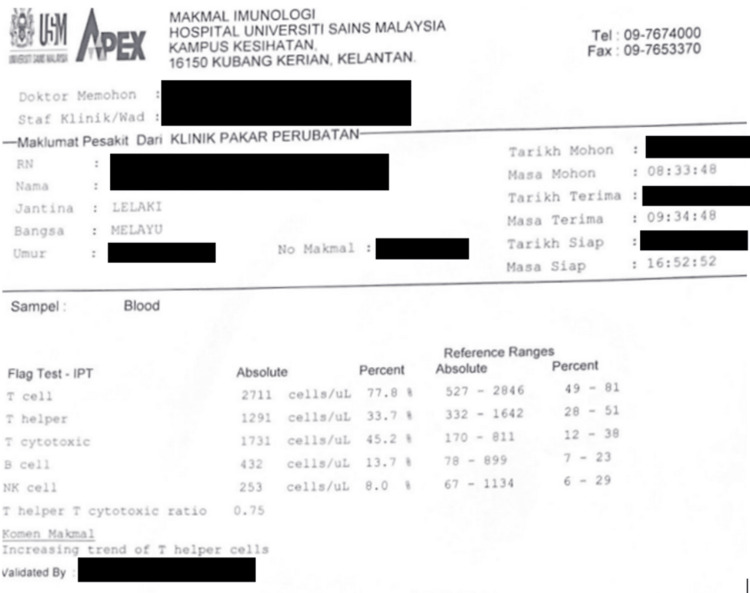
Report from immunology laboratory after HAART HAART: highly active antiretroviral therapy

Following the breaking of the bad news, the patient finally confessed to his high-risk behavior, as he had unprotected sex with multiple partners over a two-year period. The patient was then referred to an infective disease team for counseling and the start of highly active antiretroviral therapy (HAART). Blood from the patient was taken before and after HAART. Prior to HAART, blood results showed a low CD4:CD8 ratio suggestive of an immunocompromised state (Figure 7), and post-HAART treatment showed an increasing trend of T Helper cells (Figure 8). Table 2 shows a side-by-side comparison of both results.

**Table 2:** The absolute count and percentage of lymphocytes before and after HAART HAART: highly active antiretroviral therapy

Cells	Absolute (cells/µL)		Percent (%)		Reference ranges	
	Before	After	Before	After	Absolute (cells/µL)	Percent (%)
T Cell	3457	2711	78.6	77.8	527-2846	49-81
T Helper	1288	1291	29.3	33.7	332-1642	28-51
T Cytotoxic	2299	1731	52.2	45.2	170-811	12-38
B cell	370	432	8.4	13.7	78-899	7-23
NK cell	545	253	12.4	8.0	67-1134	6-29
T Helper T Cytotoxic ratio	0.56	0.75				

The one-year follow-up revealed no recurrence of the ranula or any other HIV-related new lesions.

## Discussion

There is a low prevalence of ranula associated with HIV infection. Ranula has been linked to 5.1% of HIV-positive patients in Zimbabwe [6]. Another study reported that 88.5% of HIV-positive patients in a group of 38 ranulas cases were HIV-positive [7]. A high prevalence of HIV-associated ranula has also been reported: 73.7% from 57 cases of ranula, and 63.7% from 113 referred cases of oral mucoceles [4,5]. Regardless of sample size, the results showed a high prevalence of ranula associated with HIV positivity, even though the majority of the studies were from a single center.

The link between ranula and HIV infection is still debatable, as the majority of reports come from Sub-Saharan Africa [4,6,7]. Various authors have investigated reports on the prevalence of ranula, but the link to HIV infection has not been well established [8-10].

Ranulas are most common in the first three decades of life, with a peak frequency in the first and second decades [8-10]. Few authors have raised concerns about a possible link between the high prevalence of HIV infection and plunging ranula in specific age groups; in particular, the second to fourth decade of life [4,5], and our patient is in this age range.

Despite the fact that various authors have reported its association with gender distribution, there is no concrete evidence to support the conclusion that gender plays a role in the pathogenesis of oral mucoceles and ranula [9-11]. The floor of the mouth was reported as the specific location for oral mucocele in HIV-infected patients [5], contradicting the previously reported common anatomic site as the lower lip due to its vulnerability to traumatic episodes most commonly caused by lip biting [8,12]. There was no history of trauma to the affected salivary glands, which raised clinical suspicion of HIV infection playing a role in the pathogenesis of ranula. The presence of the HIV-1 virus in the fluid content of ranula has been reported, which could explain why a ranula is one of the HIV-related salivary gland diseases [13].

Plunging ranula is a clinical entity [6]. However, it has been reported that plunging ranula is not uncommon in HIV-positive patients, with 92.1% reported in a total of 38 HIV-positive patients [5]. Ranulas are classified into two types: simple (intraoral) and plunging (cervical). The simple ranula is restricted to the mouth's floor. Because of its distinctive appearance and location, oral ranula is usually diagnosed clinically. The plunging ranula is located in the inframylohyoid compartment of the neck and may be confused with other causes of cervical swelling, especially if no oral component is present, as seen in our case initially. Oral lesions are frequently the first signs of HIV infection. The World Health Organization (WHO) classifies seven cardinal lesions as HIV-related: oral candidiasis, hairy leukoplakia, Kaposi's sarcoma, linear gingival erythema, necrotizing ulcerative gingivitis, necrotizing ulcerative periodontitis, and non-Hodgkin’s lymphomas, which are all strongly associated to HIV infection with pseudomembranous candidiasis as one of the most common lesions in an advanced HIV infection [14].

There are several methods for treating ranula, but surgery is the most common. The preferred approach and technique are variable and include marsupialization and ranula excision with or without sublingual and/or submandibular gland excision. Despite these treatments, a large number of patients experience recurrence [15]. Transoral sublingual gland excision with ranula evacuation has the lowest recurrence and complication rates (13%) [16]. The posterior-inferior displacement of the lesion in our case necessitates a combination of transcervical and transoral approaches for the complete removal of the lesion and associated affected salivary glands.

Surgeons must take universal precautions when performing surgical interventions, and there is no reason to discriminate against HIV patients with ranula. There should be equal perioperative management and follow-up for patients with and without HIV who present with plunging ranula and oral white lesions.

## Conclusions

Clinicians should master the art of taking histories as this may aid in establishing accurate diagnoses, and when there is a clinical suspicion, extra precautions should be taken. Although the pathogenesis of ranula with HIV is still unclear, there have been studies regarding the association of ranula and HIV. Clinicians should be aware that the age distribution of HIV-positive patients nearly mirrors that of plunging ranula patients (the second and third decades of life) and the presence of oral white lesions strongly suggests that the patient might have an immunocompromised state. Routine HIV testing of patients with a ranula is justified and may be recommended, especially for young patients with other intraoral suspicious lesions as in our case the patient presented with bilateral scrapable white lesions. Excision of the ranula and the sublingual gland is suggested in order to avoid recurrence. Finally, early HIV diagnosis and treatment with HAART may prevent the progression of HIV to AIDS.

## References

[1] Kinshuck AJ, Schober M, Kokai G, Clarke R (2012). Oral ranula in an HIV-positive patient: case report and literature review. BMJ Case Rep.

[2] Meer S (2019). Human immunodeficiency virus and salivary gland pathology: an update. Oral Surg Oral Med Oral Pathol Oral Radiol.

[3] El Howati A, Tappuni A (2018). Systematic review of the changing pattern of the oral manifestations of HIV. J Investig Clin Dent.

[4] Kamulegeya A, Okello SM (2012). Ranulas: possible signs for HIV/AIDS? 1 year Ugandan descriptive study. Acta Odontol Scand.

[5] Syebele K, Munzhelele TI (2015). Oral mucocele/ranula: another human immunodeficiency virus-related salivary gland disease?. Laryngoscope.

[6] Chidzonga MM, Mahomva L (2007). Ranula: experience with 83 cases in Zimbabwe. J Oral Maxillofac Surg.

[7] Butt F, Chindia M, Kenyanya T, Gathece L, Rana F (2010). An audit of ranulae occurring with the human immunodeficiency virus infecton. J Oral Maxillofac Pathol.

[8] Chi AC, Lambert PR 3rd, Richardson MS, Neville BW (2011). Oral mucoceles: a clinicopathologic review of 1,824 cases, including unusual variants. J Oral Maxillofac Surg.

[9] Mínguez-Martinez I, Bonet-Coloma C, Ata-Ali-Mahmud J, Carrillo-García C, Peñarrocha-Diago M, Peñarrocha-Diago M (2010). Clinical characteristics, treatment, and evolution of 89 mucoceles in children. J Oral Maxillofac Surg.

[10] Re Cecconi D, Achilli A, Tarozzi M, Lodi G, Demarosi F, Sardella A, Carrassi A (2010). Mucoceles of the oral cavity: a large case series (1994-2008) and a literature review. Med Oral Patol Oral Cir Bucal.

[11] Liu Y, Wang L, Zhu Y, Lan L, Wang D (2023). High self-healing rate of oral ranula: a prospective study. Innovation Medicine.

[12] Bowers EM, Schaitkin B (2021). Management of mucoceles, sialoceles, and ranulas. Otolaryngol Clin North Am.

[13] Syebele K, Bütow KW, Webber L, Manda SO (2011). Quantification of HIV-1 viral load in the fluid of ranulas in HIV-positive patients. Oral Surg Oral Med Oral Pathol Oral Radiol Endod.

[14] Rafat Z, Sasani E, Salimi Y, Hajimohammadi S, Shenagari M, Roostaei D (2021). The prevalence, etiological agents, clinical features, treatment, and diagnosis of HIV-associated oral candidiasis in pediatrics across the world: a systematic review and meta-analysis. Front Pediatr.

[15] Kolomvos N, Kalfarentzos E, Papadogeorgakis N (2019). Surgical treatment of plunging ranula: report of three cases and review of literature. Oral Maxillofac Surg Cases.

[16] Patel MR, Deal AM, Shockley WW (2009). Oral and plunging ranulas: What is the most effective treatment?. Laryngoscope.

